# Eating Competence Associated with Food Consumption and Health Outcomes among Brazilian Adult Population

**DOI:** 10.3390/nu12103218

**Published:** 2020-10-21

**Authors:** Fabiana Lopes Nalon de Queiroz, Eduardo Yoshio Nakano, Raquel Braz Assunção Botelho, Verônica Cortez Ginani, André Luiz Fernandes Cançado, Renata Puppin Zandonadi

**Affiliations:** 1 Department of Nutrition, Faculty of Health Sciences, Campus Universitário Darcy Ribeiro, University of Brasília, Distrito Federal 70910-900, Brazil; raquelbabotelho@gmail.com (R.B.A.B.); vcginani@gmail.com (V.C.G.); 2 Department of Statistics, University of Brasilia, Brasilia, DF 70910-900, Brazil; eynakano@gmail.com (E.Y.N.); acancado@unb.br (A.L.F.C.)

**Keywords:** eating competence, health outcomes, validation, questionnaire, food consumption

## Abstract

This study aimed to associate Eating Competence (EC) with food consumption and health outcomes in the Brazilian adult population. Researchers developed a questionnaire to associate EC with sociodemographic information, health outcomes, and food consumption. Data on body weight and height was referred to by participants in the questionnaire, and body mass index (BMI) was calculated and classified. A question to evaluate the perception of body size was included. After constructing the questionnaire items, content validation and semantic evaluation were performed following the Delphi method with a group of judges composed of 26 health professionals. The judges evaluated the sociodemographic information, health outcomes, and food consumption items associated with the eating competence instrument (previously validated in Brazilian-Portuguese). The final version of the questionnaire was composed of 33 items. Our results confirmed good reliability, responsiveness, and internal consistency. A total of 1810 Brazilians answered the questionnaire. Most of the participants were female, up to 40 years old, with a high education level and high income. Most of the participants did not report diabetes or hypertension. The EC total score did not differ among males and females. Individuals up to 40 years old presented a lower total score. The increase in educational level and income also increased EC total score. Excess weight individuals showed lower EC compared to the normal weight/underweight. Individuals who consumed fruits and/or vegetables five or more days/week presented the best scores for total EC.

## 1. Introduction

Eating competence (EC) is an attitudinal and behavioral concept based on The Satter Eating Competence Model (ecSatter). It is not focused on nutrients, portion size, or food groups, but rather on enjoying food and eating, paying attention to the variety in the diet, attending to signals of hunger and satiety, and preparing meals and snacks regularly with some attention to nourishing food and the environment in which it is consumed [1]. Diet quality and health are associated with EC, as many studies show that individuals considered competent eaters tend to present higher diet quality [2,3] and healthier eating behaviors [4,5]. Competent eaters also show less disordered emotional and uncontrolled eating, greater weight satisfaction [6,7,8,9], and better sleep quality [10,11,12]. Furthermore, competent eaters are physically more active [13], have lower blood pressure and dyslipidemia [14], less overweight/obesity [6,7], and have more positive attitudes and behaviors toward food and eating [9,15].

Considering that eating is a complex process involving learned behavior, social expectations, acquired tastes, attitudes, and feelings about food and eating [1], the ecSatter model was proposed as a biopsychosocial approach to eating behavior, including four components: Eating Attitudes (having a positive, relaxed, and flexible interest in food and eating); Food Acceptance (being interested in food and having the capacity to accept and like a wide variety of foods, including new foods); Internal Regulation (being naturally attentive to the internal signals of hunger, appetite, and satiety to guide how much to eat, supporting stable body weight); and Contextual Skills (having resources to manage the food context, meal planning and the permission to eat adequate amounts of preferred food at predictable times) [1,16].

EC, with its four components, can be measured by using the Satter Eating Competence Inventory (ecSI™2.0). It is a reliable [17] instrument, validated for American adults [6,7,18], composed of 16 items that classify people into two groups: eating competent (EC) and not eating competent (not EC). It allows researchers and educators to follow intervention outcomes and explore the eating competence construct [19]. The ecSI2.0™ is translated into Arabic, German, Japanese, Finnish, and Spanish [20]. It was recently translated into the Brazilian-Portuguese language, and its reproducibility was verified with a sample of the Brazilian adult population [21]. EC and its association with food consumption and health outcomes have not been previously studied in the Brazilian population. Considering that a poor quality diet, excess weight, and other chronic diseases have been increasing among Brazilians [22], the EC knowledge could help health promotion in the Brazilian population, supporting professionals and the government to develop strategies and public policies on diet, nutrition, and health. Therefore, this study aimed to associate EC to food consumption and health outcomes (body mass index, diabetes, and hypertension) in the Brazilian adult population.

## 2. Materials and Methods

The Ethics Committee of Santa Marta’s Institute of Teaching and Research (Federal District/Brazil) approved this cross-sectional study (CAAE 24415819.2.0000.8101), following the guidelines established by the Declaration of Helsinki.

Researchers used a validated Brazilian-Portuguese version of the ecSI2.0™ [21] to develop a questionnaire to associate EC with sociodemographic information, health outcomes, and food consumption. After the questionnaire’s validation, it was applied to a nationwide sample of the Brazilian adult population.

### 2.1. Development of a Questionnaire Regarding Sociodemographic Data, Health Outcomes, and Food Consumption

In addition to the Brazilian-Portuguese version of the ecSl2.0 (ecSl2.0™BR), sociodemographic questions (gender, age, income, schooling level, and housing area) were included using the questions from the Brazilian National Institute of Geography and Statistics (IBGE) as a reference [23]. The referred body height and weight questions were used to estimate the body mass index (BMI) and classify the sample. Classification followed the international BMI cut-offs for adults (BMI: Low-weight: <18.5 kg/m^2^; Normal weight: 18.5–24.9 kg/m^2^; Overweight: 25.0–29.9 kg/m^2^; Obesity: ≥30 kg/m^2^) [24,25]. A question to evaluate the perception of body size (oversize, appropriate size, and undersize) was included [26].

The consumption of healthy or unhealthy food was investigated using The Brazilian Food Questionnaire (VIGITEL) [22] recommended by the Brazilian Ministry of Health. The VIGITEL is a large population study conducted annually since 2006, and it is part of a set of actions aimed at the control of chronic non-communicable diseases in Brazil. It is based on models used to monitor risk factors for chronic diseases and has been improved over the years, and the latest version was published in 2020. The dietary intakes evaluated by the VIGITEL questionnaire includes questions on how often respondents consume beans, fruits, vegetables, milk, meats, processed foods, and sugary and non-sugary soft drinks. The VIGITEL is already validated for online use [27].

After constructing the questionnaire items, content validation and semantic evaluation were performed following the Delphi method [28]. Therefore, 30 health professionals were contacted by email to participate as judges for content validation and semantic evaluation. Twenty-six of them agreed to participate in this phase to compose the group of judges. Following their consent, participants received an email with a link to the Brazilian-Portuguese questionnaire placed on the Google Forms^®^ platform. The ecSI2.0™BR was not included for judgment once it was already approved by the Needs Center [20]. Therefore, the questionnaire was sent to the judges to evaluate the sociodemographic, health outcomes, and food consumption items, considering EC’s context.

The first page of the questionnaire had an orientation letter explaining the evaluation criteria of the items’ clarity and relevance. The judges rated the questions to perform the content validation on a five-point Likert Scale from “I totally disagree with the item”; to “I fully agree with the item.” For semantic evaluation, the judges were asked to evaluate each item regarding its clarity using a scale from “I did not understand it at all”; to “I understood it perfectly and had no questions.” Answers from 0 to 3 indicate insufficient understanding, and a new version of the item is required [29]. When applicable, they made suggestions to improve the questionnaire regarding comprehension and clarity. The judges’ group was composed of health professionals in the nutrition field, medicine, biology, and psychology, with a minimal Master of science degree (MSc 30.8%; Ph.D. 46.2%; postdoc 23.1%).

The mean score for each item of the questionnaire concerning content validation and semantic evaluation was calculated with the judges’ answers. The degree of agreement among them was evaluated through the Kendall (W) coefficient of concordance (ranging from 0 to 1). W-value ≥ 0.66 is considered high and indicates that the judges applied the same evaluation [30,31]. The criteria used for the item’s approval was a minimal of 80% of judges’ agreement (W-value ≥ 0.8) [29,30,31].

### 2.2. National Study to Associate EC with Food Consumption and Health Outcomes in the Brazilian Population

After the agreement among the judges, the final version of the questionnaire (including the ecSI2.0™BR, food consumption, and health outcome items) was applied in a convenience sample of 32 individuals answering twice (58.6 ± 43.2 h interval) to confirm the reproducibility of the questionnaire [21]. The reproducibility of health outcomes was not evaluated because it is an internal characteristic that does not change its perception in a short period.

After the reproducibility confirmation (ICC and Kappa > 0.6), the questionnaire was applied in a nationwide sample of the Brazilian adult population. It was a convenience sample since participants received the invitation through email, messaging apps, and social networks. When starting to answer the questionnaire using the SurveyMonkey^®^ tool, an online survey platform, participants received the consent form approved by the research ethics committee. Those who agreed to participate and met the inclusion criteria (age ≥ 18 years; live in Brazil) were included in the study.

### 2.3. Statistical Analysis

The questionnaire’s responsiveness was verified by the floor and ceiling effects. Floor effect is observed when ecSI2.0™BR (and its four components) produces a score equal to zero, and the ceiling effect occurs when the instrument (and its four components) reaches maximum values. Cronbach’s alpha coefficient evaluated the internal consistency of the ecSI2.0™BR. The scores of the ecSI2.0™BR (and its components) were described in terms of means, standard deviations (SD), medians, and interquartile range. Student’s *t*-test and Analysis of Variance (ANOVA) followed by Tukey’s post-hoc tests were used to compare the scores of ecSI2.0™BR with interest variables. The Kolmogorov-Smirnov test verified the normality assumption. Results of categorized eating competence (EC ≥ 32) were described in terms of frequencies and percentages, and Person chi-squared tests verified its association with the variables of interest. All tests were performed considering bilateral hypotheses and a 5% significance level. The analyzes were performed using IBM SPSS (IBM SPSS Statistics for Windows, IBM Corp, Armonk, NY, USA) version 22.

## 3. Results

**Figure 1:**
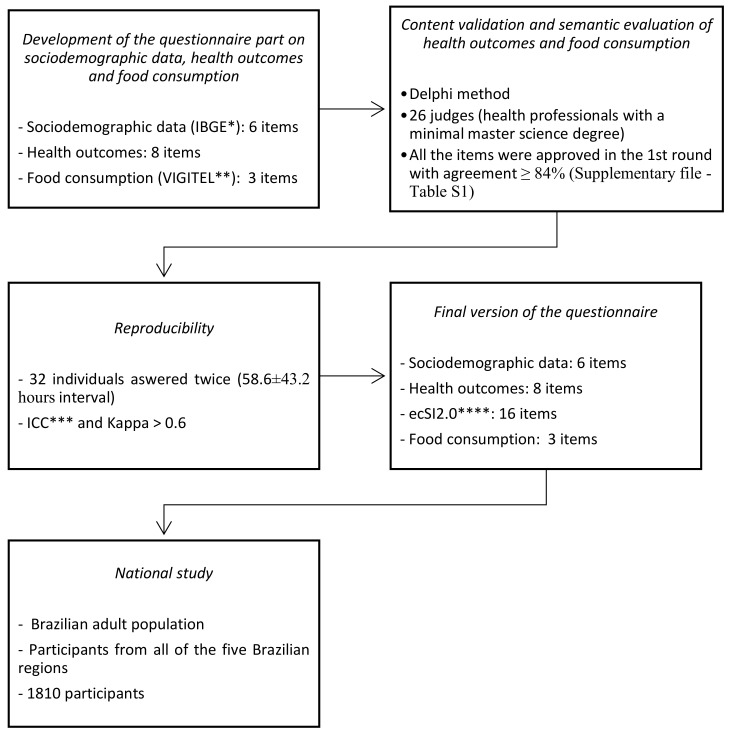
Flowchart of the steps to construct and validate the final version of the questionnaire and national application. * IBGE: Brazilian Institute of Geography and Statistics; ** VIGITEL: Brazilian Surveillance System of Risk and Protection Factors for Chronic Diseases by Telephone Inquiry”; *** ICC: Intraclass correlation coefficient; **** ecSI: Satter Eating Competence Inventory.

The steps of constructing and validating the complete questionnaire, composed of the ecSI2.0™BR, sociodemographic data, health outcomes, and food consumption, are described in Figure 1. The final version of the questionnaire was composed of 33 items (Appendix A).

The complete questionnaire (Appendix A) was constructed considering the socioeconomic data, health outcomes, EC, and food consumption. All the items were approved in the first round by the judges (Figure 1), indicating that questions were considered necessary and easy to comprehend.

The reproducibility (test-retest reliability) of the questionnaire, conducted with 32 Brazilian adults, showed a total ICC score > 0.8, indicating excellent reproducibility. Considering the entire questionnaire, none of their parts presented significant divergence among the evaluators (*p* < 0.001).

Table 1 presents data for internal consistency and ecSI2.0™BR scores. The possible range was from 0 to 48 for the total eating competence score, 0–18 for eating attitudes, 0–15 for contextual skills, 0–9 for food acceptance, and 0–6 for internal regulation, as recently suggested by Godleski [19] and approved by the NEED’s Center [20]. The ecSI2.0™BR showed both good internal consistency (Cronbach’s alpha coefficient was 0.868 for the ecSI2.0™BR total scale, 0.793 for Eating Attitudes, 0.704 for Food Acceptance, 0.543 for Internal Regulation, and 0.815 for Contextual Skills) and responsiveness (floor and ceiling effects ≤ 16.6% for all components and ≤ 0.5% for the entire questionnaire).

### Questionnaire in the Brazilian Population

The questionnaire was available online from December 2019 to March 2020 (before the Sars-Cov-2 pandemic period in Brazil). From 1922 individuals who accessed the questionnaire, a total of 1810 (94.17%) answered it. Table 2 shows the sociodemographic characteristics of the participants and the EC scores by these characteristics. Most of the participants were female (*n* = 1353; 74.75%), up to 40 years old (*n* = 975; 53.6%), with a high level of education (*n* = 882; 48.72% were graduates) and family income > R$ 5000 (*n* = 1239; 67.9%). Regarding weight, 48.06% of the sample (*n* = 869) presented normal weight, and 49.44% (*n* = 895) excess weight (considering overweight and obesity together). Despite that, the body size perception was reported as oversize by 56.13% (*n*= 1016). Most of the participants did not report diabetes (*n* = 1729; 95.52%) or hypertension (*n* = 1567; 86.57%). The consumption of fruits was reported most frequently as five or more days/week (*n* = 910; 50.3%) as well as vegetables (*n* = 1196; 66.11%). The consumption of artificial juice and soda was reported as rarely by more than half of the studied population (*n* = 1032; 57.04%).

The EC total mean score was 30.19 ± 8.90 (Table 1) and did not differ among males and females (29.94 ± 8.52 vs. 30.26 ± 9.02; *p* > 0.05) and among those who had diabetes or hypertension or not (*p* > 0.05). Individuals up to 40 years old presented a lower total score (as well as in the components of eating attitude, internal regulation, and contextual skills) compared to the ones older than 40 years (28.90 ± 9.18 vs. 31.69 ± 8.31; *p* < 0.05). For food acceptance, the score did not differ among age groups. The increase in educational level and income also increased EC total score, food acceptance, and contextual skills. However, it was not different considering eating attitude and internal regulation (Table 2).

Obese individuals showed lower EC (26.09 ± 8.98) compared to the overweight (29.42 ± 9.21) and the normal weight (32.10 ± 8.02). Individuals who perceived their body size as appropriate presented the best total score for EC and those who reported being oversized presented the worst scores (33.63 ± 7.56 vs. 27.7± 9.02; *p* < 0.000). Individuals who consumed fruits and/or vegetables five or more days/week presented the best scores for total EC and each component, different from those consuming artificial juice or soda for five or more days/week who presented the worst total scores (Table 2).

Eating competent individuals (EC ≥ 32) were mostly individuals over 40 y/o; graduated; income higher than 5000 reais; with a good perception of body size; with adequate fruits and vegetables (FV) consumption and low consumption of artificial juices and soda (Table 2).

## 4. Discussion

This study is the first one in which the ecSI2.0™BR instrument has been used to assess Brazilian adults’ EC. It was performed with a sample of 1810 Brazilian adults, mostly females, with a high level of education and income, who answered a questionnaire regarding sociodemographic data, health outcomes, food consumption, and EC.

The online application of a self-administered instrument was chosen because it is less costly for the research and less invasive for the participants than a face-to-face interview, which can also place difficulties in reaching a more diverse population due to geographical limitations [32]. Data from the Brazilian Institute of Geography and Statistics (IBGE) shows that 3 out of 4 Brazilians have internet access. The number of cellphone possession, a primary tool used to access the internet, increased from 92.6% to 93.2% [33]. Therefore, the online self-reported method shows efficiency for data collection, the possibility to reach a more significant number of participants, and a positive impact on cost.

A semantic evaluation and content validation were performed to evaluate the instrument’s reliability and ensure it is straightforward and easy to understand. Twenty-six health professionals approved the instrument in the first round of judgment. Our results confirmed good reliability, responsiveness, and internal consistency, showing Cronbach’s alpha > 0.70 (Cronbach’s alpha of 0.868 for the ecSI2.0™BR total scale; 0.793 for Eating Attitudes; 0.704 for Food Acceptance; and 0.815 for Contextual Skills). Only the Internal Regulation component presented lower Cronbach’s alpha (0.543), but the total questionnaire showed good internal consistency (Table 1). A previous study with a smaller sample of Brazilian adults (*n* = 662, 74.9% female) using ecSI2.0™BR showed similar results as Cronbach’s alpha coefficient was 0.869 for the ecSI2.0™BR total scale; 0.793 for Eating Attitudes; 0.527 for Internal Regulation; 0.728 for Food Acceptance; and 0.822 for Contextual Skills [21]. The internal regulation domain is composed of only two statements. Considering that the Cronbach´s alpha value is affected by the number of items, both studies’ low value may be explained by its small scale [34]. Tanja et al. (2020) measured EC using a translated Finnish version of the ecSI2.0™ with a sample of 3147 Finnish adults aged 18–74 at an increased risk for type 2 diabetes. They found a Cronbach’s alpha coefficient of 0.83 for the total ecSI2.0™; 0.77 for eating attitudes; 0.62 for food acceptance; 0.73 for internal regulation; and 0.76 for contextual skills. In our study, the ecSI2.0™BR presented good responsiveness, showing total floor and ceiling effects ≤ 0.5% (<16.6% if we consider the four components). The items of the questionnaire showed reliable internal consistency. Therefore, the questionnaire presents good measures of reproducibility.

Using the validated cut-off value of ≥32 [6,20], 47.73% of the sample were classified as competent eaters. This proportion is well in line with previous results, in which 45–59% of adult populations were considered competent eaters [2,3]. Depending on the population (age group, income, and the presence of diseases), the proportion of competent eaters may vary between 18% and 53% [3,6,7,13,14,35,36]. In the present study, EC individuals (EC total score ≥ 32) were mostly the individuals older than 40 years; graduated; with a high income; who reported a good perception of body size; with adequate consumption of fruits and vegetables and low consumption of artificial juice and soft drinks. A previous study with a general audience of 832 American adults (mean age 36.2 ± 13.4 y/o; 78.7% female) showed similar results as EC individuals were also older, reported lower BMI and incidence of overweight, and were less dissatisfied with their weight [6].

Considering the four EC components, the contextual skills showed a positive association with age, education level, income, BMI, and food consumption. A recent Brazilian study confirmed the importance of contextual food abilities showing that parents’ cooking skills confidence protects their children from eating more industrialized foods promoting healthy eating [37]. In the present study, the food acceptance component showed a positive association with food consumption and the perception of body size, which is expected considering the ecSatter definitions for these components of EC [1]. Food preparation skills have been largely studied and positively related to diet variety, increasing diet quality [38,39,40,41]. In this sense, the development of cooking skills by hands-on cooking classes has become an emerging way to promote healthy eating and empower individuals and families to incorporate healthy behaviors with a greater variety of foods [42]. It reinforces the contextual skills and food acceptance components provided in the ecSatter model [1] and suggests that learning food preparation skills enhances EC and potentially dietary variety and quality.

In our study, the internal regulation component showed an association with BMI, perception of body size, and food consumption. It is well in line with the definition of internal regulation proposed by the ecSatter model [1,16]. Body weight dissatisfaction is predictive of poor EC, not only because individuals who are dissatisfied with their body weight have negative attitudes about food and eating, but they may also be less likely to try new foods and plan and prepare regular meals for themselves [9]. A lack of the internal regulation component is linked to the inability to identify the sensations related to hunger and satiety and the presence of bulimic thoughts and feelings of uncontrolled hunger [6]. In our sample, eating attitudes were not related to schooling level or income, but related to age, gender, perception of body size, and food consumption. Eating attitude is a crucial component of the EC. Food and eating can be associated with ambivalent feelings, ranging from pleasure and enjoyment to worry about weight gain, body appearance, and health effects. A New Zealand study (*n* = 294; 72% female; with high education level) about eating behaviors and body weight found a significant relationship between wanting to lose weight and associating chocolate cake with guilt. Furthermore, participants who associated chocolate cake with guilt were less successful at maintaining their weight or losing weight over three months than those who associated chocolate cake with celebration [43]. The finding that associating chocolate cake with celebration was related to more successful weight maintenance and it is under the eating attitude proposed in the ecSatter model [1,16], reinforcing the importance of eating attitude in food choice and health. 

In the present study, the sample was predominantly composed of females (74.87%), indicating that women tend to be more concerned about health in general and participate in health surveys more than men [30,44]. Considering the total EC score, there is no difference between females and males (*p* = 0.518). This result is in line with those reported by Lohse et al., who noticed that gender did not predict EC among the US adults. However, this finding is opposite to that reported by other studies when males showed better EC than females [8,9]. The association between EC and gender seems to vary depending on the age group. Brown et al. [8] found that among college students (*n* = 343 and age 18–20 years old; *n* = 180 and age 21–26 uears old), males were more competent eaters than females [8]. On the other hand, Tanja et al. [26], using a preliminary Finnish translation of the ecSI2.0 for evaluating presumed EC and its association with food selection, meal patterns, and related psychobehavioral factors among 10–17-year-old adolescents (*n* = 976), showed that girls were more often presumably competent eaters than boys [26]. In the present study, considering the four components, males presented a higher score for eating attitudes (*p* = 0.000) and internal regulation (*p* = 0.010), suggesting that Brazilian males have a more comfortable or relaxed approach to eating than Brazilian females. Females had a better score regarding food acceptance (*p* = 0.000) and contextual skills (*p* = 0.238). In Brazil, it is expected that females have better resources to manage the food context as they play a central role in family food purchasing and preparation, reporting sole responsibility for household food decisions [23,45].

Our results showed that EC in the Brazilian population was directly proportional to education and income level. EC is usually related to income as low-income persons have less favorable dietary patterns and are also less eating competent [15,46]. The ability to be eating-competent is affected by food insecurity and/or income restrictions, as having worries about money for food is associated with a lack of eating competence [6]. Lohse et al., studying a low-income sample of females (*n* = 149; 56% white; 64% food secure; 86% was 18–50 y/o), found that dietary patterns, nutrient intakes, and overall diet quality were increased among those with better scores for total EC [2].

Most of our sample was classified as normal weight (40.86%), followed by overweight (32.68%) and obesity (16.81%) according to the BMI classification. According to the last VIGITEL data [22] in the Brazilian adult population, the frequency of overweight is 55.4%, being higher among males (57.1%) than females (53.9%); and the frequency of obesity is 20.3%, with no differences between males and females. It is well documented that, among females, the frequency of overweight and obesity decreases significantly with increased education [22]. In the present study, participants were more educated than the general Brazilian population. The mean age was relatively high, associated with higher EC [2,6], explaining the differences. We found that eating competent individuals had smaller BMI than non-eating competent ones. Our results are well in line with previous publications that have reported an inverse association of EC and BMI [6,7,9,10,18,35]. In a previous one-year weight loss intervention with premenopausal, mostly college-educated American females, EC increased as BMI decreased among obese individuals [35]. There is some evidence that improving contextual skills and positive eating attitudes in weight management interventions help with losing weight [35], suggesting that enhancing these EC components may contribute to weight loss. 

According to the BMI classification, 49.44% of the sample presented an excess weight, but 56.13% reported the perception of body size as oversize. The ecSatter model emphasizes satisfaction with bodyweight [1]. In line with this, in the present study, individuals who more often perceived their body size as appropriate showed higher EC scores than those who reported being oversized (EC total score = 33.63 ± 7.56 vs. 27.7 ± 9.02; *p* < 0.000). Body dissatisfaction is a risk factor for overweight and eating disorders [6]. Considering the four components of EC, there were statistical differences (*p* < 0.05) between those who reported being at an appropriate size from those who reported being oversized (Table 2). Previous studies regarding EC showed a positive association between EC and body satisfaction [7,8,9,47]. Clifford [9] studied a convenience sample of 1720 American college students to determine which factors were more predictive of EC, their body mass index (BMI), or their attitude about their weight. The author found that weight satisfaction and desire to lose weight were better predictors of EC than BMI [9].

Only 4.47% of our sample reported diabetes, and 13.42% reported hypertension, and EC was not associated with these non-communicable diseases. However, previous studies with different populations showed associations between EC, BMI, and risk for type 2 diabetes and other cardiovascular risk diseases as hypertension [3,14,48]. For example, Psota et al. [14] reported that competent eaters have a more healthful cardiovascular risk profile, including lower blood pressure (*n* = 48; 21 to 70 years of age). A recent Finnish study with 3.147 adults (18–74 years at elevated risk for type two diabetes) showed that EC competent individuals (only 37% of the sample) showed better diet quality [48]. Among elderly Spanish individuals (*n* = 638; at high risk for cardiovascular disease and nearly 55% had diabetes mellitus), EC had a positive association with higher healthier dietary habits and fewer cardiovascular risk factors [3]. It indicates an association between EC and these diseases and that EC improvements may help prevent diabetes and other chronic diseases. Our sample showed fewer individuals with diabetes and hypertension than the general population in Brazil. The results may be explained by the age and schooling characteristics of the sample. According to the last VIGITEL, the frequency of diabetes among adults is 7.4%, and hypertension is 24.5%. Both increase with age and decrease with the level of education [22].

The consumption of fruits and vegetables (FV) is used as a healthier indicator, and it is considered adequate when the regular frequency is at least five times per week [49]. According to the last VIGITEL data [22], only 34.3% of the Brazilian adult population have ingestion of FV five times per week, being lower among males (27.9%) than females (39.8%), and this frequency tends to increase according to age and schooling for both genders. Better results were found in our study showing that 50.3% of the sample consume fruits five or more days/week, and 66.1% reported consuming vegetables five or more days/week. The frequency of FV consumption was directly proportional to the total EC scores and its four components, indicating that EC is associated with greater consumption of FV, which is presumably related to better health and seems to protect against overweight. The results on food consumption in this study are well in line with previous studies where EC was associated with diet quality [7,15,26,46], including greater adherence to a Mediterranean diet [3].

As expected, in the present study, EC was inversely associated with artificial juice or soda consumption. The regular intake of soft drinks was used as an indicator of unhealthy eating habits. Their consumption is associated with higher energy intake, elevated BMI, and increased risk of medical outcomes [23]. Furthermore, more than being a marker of poor nutrition, regular intake of soft drinks has been related to lower intake of fruits and fiber, and higher intake of fast foods and carbohydrates with a higher glycemic index [50]. The average percentage of individuals regularly consuming artificial juice and soda was 9.06% in our study. More than half of the studied population (57.04%) reported consuming artificial juice and soda as “rarely”. According to the last VIGITEL, the consumption frequency of soft drinks five or more days a week in Brazilian adults is 15%, higher among males than females. For both genders, the frequency of consumption decreases according to age and is more elevated in the intermediate educational level [22]. Industrialized drinks are considered highly industrialized foods by the Dietary Guidelines for the Brazilian population. They should be avoided because they are rich in sugar and/or sweeteners and chemical additives [51]. The intake of sugary drinks, a marker of negative diet quality, was low in the studied sample, which can be considered a positive result observed in our sample, compatible with the health data presented.

Nonetheless, despite these positive results in our study, a large proportion of the Brazilian adult population does not achieve the FV daily intake, according to the WHO recommendations [22]. It may be linked to the fact that overall diet quality measures reveal poor dietary patterns among the low-income Brazilian population [22]. Therefore, strategies aimed at increasing EC could improve FV intake and target the Brazilian adult population to improve diet quality and health.

This study’s significant strengths include using a validated tool for the Brazilian population and already used in previous studies, allowing us to make comparisons. Another strength is recruiting a high number of individuals (*n* = 1810) from the entire country to participate, enabling us to generate more consistent data in this area.

As this is the first study using the ecSI2.0™BR among Brazilian adults, the results should be interpreted cautiously. A limitation of this study is that our sample had a higher proportion of female respondents, and male were underrepresented, limiting the generalizability of the results to the population. There was also a selection bias regarding the socioeconomic level of the sample. According to a Brazilian Institute of Geography and Statistics (IBGE) census, approximately half of the Brazilian population 25 years of age and younger have less than eighth-grade education [23]. However, 48.72% of our respondents were graduates, which is not representative of the Brazilian population and indicates that the associations presented here need to be tested among the socioeconomically disadvantaged Brazilian population. It is essential to highlight that our sample size was affected by the Sars-Cov-2 pandemic. At the beginning of the pandemic period, we interrupted the data collection since EC could be affected by life changes. We decided not to use any promotional material for the survey because it was an unexpectedly sensitive time, marked by fears and uncertainties. 

## 5. Conclusions

Eating competence, as defined and measured by the ecSI2.0™BR, seems to be a useful concept in Brazilian adults. Overall, as an indicator of positive eating-related behavior and attitude, EC was associated with health-promoting food consumption and health outcomes. The EC total score did not differ among males and females. Individuals up to 40 years old presented a lower total score. The increase in educational level and income also increased EC total score. Excess weight individuals showed lower EC compared to the normal weight/underweight. Individuals that consume fruits and/or vegetables five or more days/week presented the best scores for total EC. Measuring the components of EC is relevant in the context of promoting nutritional health. Therefore, in the future, enhancing EC by innovative strategies based on emerging behavioral theories can improve nutritional education policy (or programs) and individual intervention. 

## Figures and Tables

**Table 1:** The ecSI2.0™BR scores, responsiveness, and internal consistency of the questionnaire (*n* = 1810, Brazil).

	Mean (DP)	Median (Q1–Q3)	Range	Floor Effect (%)	Ceiling Effect (%)	Cronbach’s Alpha
Eating attitude	12.06 (3.91)	13 (9–15)	0–18	0.2%	6.3%	0.793
Food acceptance	5.03 (2.42)	5 (3–7)	0–9	3.8%	8.2%	0.704
Internal regulation	3.85 (1.53)	4 (3–5)	0–6	2.9%	16.6%	0.543
Contextual skills	9.26 (3.70)	10 (7–12)	0–15	0.8%	6.9%	0.815
Total	30.19 (8.90)	31 (24–37)	1–48	0%	0.5%	0.868

**Table 2:** Sub-scores of the ecSI2.0 scale subcategorized by sociodemographic variables and health and consumption characteristics (*n* = 1810 − Brazil).

	Eating Attitude	Food Acceptance	Internal Regulation	Contextual Skills	Total	ecSI2.0™BR ≥ 32
	Mean (SD)	Mean (SD)	Mean (SD)	Mean (SD)	Mean (SD)	Freq. (%)
Gender *						
Female (*n* = 1353)	11.87 (4.00) ^A^	5.15 (2.42) ^A^	3.80 (1.51) ^A^	9.45 (3.66) ^A^	30.26 (9.02) ^A^	658 (48.6%) ^A^
Male (*n* = 454)	12.61 (3.61) ^B^	4.64 (2.39) ^B^	4.01 (1.55) ^B^	8.69 (3.75) ^A^	29.94 (8.52) ^A^	206 (45.4%) ^A^
*p*	0.000	0.000	0.010	0.238	0.518	0.233 ***
Age *						
Up to 40 years (*n* = 975)	11.58 (4.09) ^A^	5.06 (2.48) ^A^	3.68 (1.56) ^A^	8.58 (3.72) ^A^	28.90 (9.18) ^A^	413 (42.4%) ^A^
More than 40 years (*n* = 835)	12.62 (3.62) ^B^	4.99 (2.35) ^A^	4.04 (1.47) ^B^	10.04 (3.52) ^B^	31.69 (8.31) ^B^	453 (54.3%) ^B^
*p*	0.000	0.542	0.000	0.000	0.000	0.000 ***
Schooling level **						
High School (*n* = 159)	11.84 (4.33) ^A^	4.67 (2.51) ^A^	4.10 (1.69) ^A^	8.60 (4.06) ^A^	29.21 (9.52) ^A^	71 (44.7%) ^A^
Undergraduate (*n* = 769)	12.05 (3.88) ^A^	4.85 (2.43) ^AB^	3.86 (1.54) ^A^	8.80 (3.79) ^A^	29.56 (8.96) ^AB^	344 (44.7%) ^A^
Graduate (*n* = 882)	12.10 (3.87) ^A^	5.24 (2.38) ^B^	3.79 (1.49) ^A^	9.77 (3.48) ^B^	30.90 (8.67) ^B^	451 (51.1%) ^B^
*p*	0.735	0.001	0.064	0.000	0.003	0.024 ***
Family income ^+,^**						
Up to R$ 3000 (*n* = 325)	11.90 (4.12) ^A^	4.90 (2.38) ^A^	3.85 (1.55) ^A^	8.63 (3.93) ^A^	29.27 (9.23) ^A^	145 (44.6%) ^A^
R$ 3001 to R$ 5000 (*n* = 255)	11.85 (3.48) ^A^	4.57 (2.47) ^A^	3.82 (1.66) ^A^	8.73 (3.70) ^A^	28.98 (9.39) ^A^	112 (43.9%) ^A^
R$ 5001 to R$ 10,000 (*n* = 417)	11.98 (4.07) ^A^	5.08 (2.36) ^B^	3.83 (1.53) ^A^	9.17 (9.79) ^AB^	30.06 (9.13) ^AB^	190 (45.6%) ^AB^
R$ 10,001to R$ 20,000 (*n* = 455)	12.08 (4.06) ^A^	5.10 (2.42) ^B^	3.84 (3.42) ^A^	9.75 (8.02) ^B^	30.76 (8.24) ^AB^	220 (48.4%) ^AB^
More than R$ 20,000 (*n* = 358)	12.41 (3.91) ^A^	5.31 (1.32) ^B^	3.90 (3.48) ^A^	9.67 (9.21) ^B^	31.29 (8.61) ^B^	199 (55.6%) ^B^
*p*	0.361	0.004	0.973	0.000	0.003	0.014 ***
BMI kg/m^2^ **						
Low: <18.5 (*n* = 44)	12.89 (4.12) ^A^	5.07 (2.68) ^A^	4.50 (1.32) ^A^	8.34 (3.42) ^AB^	30.80 (9.79) ^AB^	20 (45.5%) ^A^
Normal: 18.5–24.9 (*n* = 869)	12.92 (3.48) ^A^	5.25 (2.38) ^A^	4.09 (1.41) ^AB^	9.83 (3.48) ^C^	32.10 (8.02)^B^	490 (56.4%) ^A^
Overweight: 25.0–29.9 (*n* = 591)	11.57 (4.07) ^B^	4.88 (2.47) ^AB^	3.73 (1.55) ^BC^	9.24 (3.68) ^AC^	29.42 (9.21)^A^	268 (45.3%) ^A^
Obesity: ≥ 30 (*n* = 304)	10.40 (4.06) ^B^	4.66 (2.36) ^B^	3.28 (1.66) ^C^	7.76 (3.93) ^A^	26.09 (8.98) ^C^	86 (28.3%) ^A^
*p*	0.000	0.001	0.000	0.000	0.000	0.000 ***
Perception of body size **						
Undersize (*n* = 64)	13.38 (3.71) ^A^	4.61 (2.50) ^A^	4.31 (1.39) ^A^	7.97 (4.00) ^A^	30.27 (9.01) ^A^	32 (50.0%) ^A^
Appropriate size (*n* = 730)	13.58 (3.19) ^A^	5.40 (2.35) ^B^	4.30 (1.31) ^A^	10.35 (3.29) ^B^	33.63 (7.46) ^B^	463 (63.4%) ^B^
Oversize (*n* = 1016)	10.88 (4.00) ^B^	4.78 (2.43) ^AB^	3.49 (1.58) ^B^	8.55 (3.76) ^A^	27.70 (9.02) ^C^	371 (36.5%) ^C^
*p*	0.000	0.000	0.000	0.000	0.000	0.000 ***
Diabetes *						
No (*n* = 1729)	12.07 (3.91) ^A^	5.03 (2.42) ^A^	3.86 (1.51) ^A^	9.24 (3.70) ^A^	30.19 (8.91) ^A^	832 (48.1%) ^A^
Yes (*n* = 81)	11.79 (3.91) ^A^	4.98 (2.48) ^A^	3.67 (1.78) ^A^	9.6 (3.63) ^A^	30.04 (8.72) ^A^	34 (42.0%) ^A^
*p*	0.529	0.849	0.350	0.384	0.878	0.307 ***
Hypertension *						
No (*n* = 1567)	12.01 (3.92) ^A^	5.06 (2.42) ^A^	3.84 (1.52) ^A^	9.19 (3.66) ^A^	30.10 (8.87) ^A^	740 (47.2%) ^A^
Yes (*n* = 243)	12.38 (3.83) ^A^	4.82 (2.40) ^A^	3.89 (1.59) ^A^	9.65 (3.92) ^A^	30.74 (9.05) ^A^	126 (51.9%) ^A^
*p*	0.169	0.153	0.618	0.071	0.292	0.190 ***
Fruit´s consumption **						
Never/rarely (*n* = 168)	11.14 (4.25) ^A^	3.42 (2.65) ^A^	3.65 (1.68) ^A^	6.20 (3.90) ^A^	24.40 (9.49) ^A^	38 (22.6%) ^A^
1 to 4 days/week (*n* = 731)	11.55 (4.05) ^A^	4.69 (2.38) ^B^	3.68 (1.55) ^A^	8.26 (3.58) ^B^	28.18 (8.86) ^B^	284 (38.9%) ^B^
5 or more days/week (*n* = 910)	12.63 (3.64) ^B^	5.59 (2.22) ^C^	4.02 (1.46) ^B^	10.62 (3.12) ^C^	32.86 (7.83) ^C^	543 (59.7%) ^C^
*p*	0.000	0.000	0.000	0.000	0.000	0.000 ***
Vegetable´s consumption **						
Never/rarely (*n* = 86)	10.38 (4.36) ^A^	2.67 (2.44) ^A^	3.59 (1.85) ^A^	6.20 (3.73) ^A^	22.85 (9.17) ^A^	13 (15.1%) ^A^
1 to 4 days/week (*n* = 527)	11.06 (3.93) ^A^	3.92 (2.28) ^B^	3.68 (1.56) ^AB^	7.50 (3.59) ^B^	26.16 (8.49) ^B^	150 (28.5%) ^B^
5 or more days/week (*n* = 1196)	12.61 (3.75) ^B^	5.68 (2.19) ^C^	3.94 (1.48) ^B^	10.25 (3.32) ^C^	32.48 (8.11) ^C^	702 (58.7%) ^C^
*P*	0.000	0.000	0.001	0.000	0.000	0.000 ***
Artificial juice or soda consumption **						
Never/rarely (*n* = 1032)	12.52 (3.73) ^A^	5.46 (2.29) ^A^	3.98 (1.45) ^A^	10.40 (3.34) ^A^	32.37 (8.26) ^A^	596 (57.8%) ^A^
1 to 4 days/week (*n* = 613)	11.39 (4.07) ^B^	4.53 (2.45) ^B^	3.66 (1.56) ^B^	8.11 (3.47) ^B^	27.69 (8.88) ^B^	226 (36.9%) ^B^
5 or more days/week (*n* = 164)	11.61 (4.05) ^B^	4.13 (2.52) ^B^	3.68 (1.76) ^B^	6.32 (3.74) ^C^	25.75 (8.81) ^C^	43 (26.2%) ^C^
*p*	0.000	0.000	0.000	0.000	0.000	0.000 ***

* Student t-test, ** ANOVA with Tukey’s post-hoc test. For each variable, the same letters comparing lines do not differ significantly, *** Pearson chi-squared test. Groups with the same letters (A, B, C) do not differ significantly, ^+^ 1.00USD = 5.56 BRL (05/05/2020). Note: The sum can be less than 1810 individuals due to the presence of missing values.

## Appendix A

### Brazilian-Portuguese Questionnaire

Parte 1: Dados sociodemográficos

Quais as iniciais do seu nome?

1. Qual o seu gênero?
  ○ Feminino
  ○ Masculino
2. Qual a sua idade? (em anos)
3. Em que estado brasileiro você mora?
4. Qual é a sua escolaridade?
  ○ Sem instrução
  ○ Fundamental incompleto
  ○ Fundamental completo
  ○ Médio incompleto
  ○ Médio completo
  ○ Superior incompleto
  ○ Superior completo
  ○ Pós graduação
5. Qual é a sua renda familiar média mensal? (em reais)
  ○ 1,00 a 500,00
  ○ 501,00 a 1.000,00
  ○ 1.001,00 a 2.000,00
  ○ 2.001,00 a 3.000,00
  ○ 3.001,00 a 5.000,00
  ○ 5.001,00 a 10.000,00
  ○ 10.001,00 a 20.000,00
  ○ 20.001,00 a 100.000
  ○ 100.001 ou mais

Parte 2: Dados de saúde

1. Quantos quilos você pesa? Se você não sabe, por favor coloque um valor aproximado.
2. Qual é a sua altura? Se você não sabe, por favor coloque um valor aproximado.
3. Em relação ao seu peso você acha que:
  ○ Está acima do peso
  ○ Está abaixo do peso
  ○ Está bem com o peso atual
4. Você tem diabetes?
  ○ Sim
  ○ Não
5. Caso tenha respondido SIM, como você trata a diabetes?
  ○ Com remédio (comprimidos e/ ou insulina)
  ○ Com dieta
  ○ Com remédios (comprimidos e/ ou insulina) E dieta
6. Você tem pressão alta (hipertensão)?
  ○ Sim
  ○ Não
7. Caso tenha respondido SIM, como você trata a pressão alta (hipertensão)?
  ○ Com remédio
  ○ Com dieta
  ○ Com remédio E dieta

Parte 3: Inventário de Competências Alimentares (ecSI2.0™BR)

Visit https://www.needscenter.org/satter-eating-competencemodel-ecsatter/eating-competence/ for permission to use the ecSI2.0™ and ecSI2.0™BR (used in Part 3 of the questionnaire).

Parte 4: Consumo de Alimentos

1. Em quantos dias da semana você costuma comer frutas?
  ○ 1 a 2 dias por semana
  ○ 3 a 4 dias por semana
  ○ 5 a 6 dias por semana
  ○ Todos os dias incluindo sábado e domingo
  ○ Quase nunca como frutas
  ○ Nunca como frutas
2. Em quantos dias da semana você costuma comer pelo menos um tipo de verdura ou legume (ex: tomate, alface, couve, cenoura, chuchu, beterraba, abobrinha, berinjela - não vale batata, mandioca ou inhame)?
  ○ 1 a 2 dias por semana
  ○ 3 a 4 dias por semana
  ○ 5 a 6 dias por semana
  ○ Todos os dias incluindo sábado e domingo
  ○ Quase nunca como verdura ou legume
  ○ Nunca como verdura ou legume
3. Em quantos dias da semana você costuma beber refrigerante ou suco artificial (caixinha, garrafa ou em pó)?
  ○ 1 a 2 dias por semana
  ○ 3 a 4 dias por semana
  ○ 5 a 6 dias por semana
  ○ Todos os dias incluindo sábado e domingo
  ○ Quase nunca bebo refrigerante ou suco artificial
  ○ Nunca bebo refrigerante ou suco artificial

## References

[1] Satter E. (2007). Eating Competence: Definition and Evidence for the Satter Eating Competence Model. J. Nutr. Educ. Behav..

[2] Lohse B., Bailey R.L., Krall J.S., Wall D.E., Mitchell D.C. (2012). Diet quality is related to eating competence in cross-sectional sample of low-income females surveyed in Pennsylvania. Appetite.

[3] Lohse B., Psota T., Zazpe I., Sorli V., Salas-salvado J., Ros E. (2010). Eating Competence of Elderly Spanish Adults Is Associated with a Healthy Diet and a Favorable Cardiovascular Disease Risk Profile 1–3. J. Nutr..

[4] Lohse B., Cunningham-Sabo L. (2012). Eating Competence of Hispanic Parents Is Associated with Attitudes and Behaviors That May Mediate Fruit and Vegetable-Related Behaviors of 4th Grade Youth. J. Nutr..

[5] Tylka T.L., Eneli I.U., Kroon Van Diest A.M., Lumeng J.C. (2013). Which adaptive maternal eating behaviors predict child feeding practices? An examination with mothers of 2- to 5-year-old children. Eat. Behav..

[6] Lohse B., Satter E., Horacek T., Gebreselassie T., Oakland M.J. (2007). Measuring Eating Competence: Psychometric Properties and Validity of the ecSatter Inventory. J. Nutr. Educ. Behav..

[7] Krall J.S., Lohse B. (2011). Validation of a measure of the Satter eating competence model with low-income females. Int. J. Behav. Nutr. Phys. Act..

[8] Brown L.B., Larsen K.J., Nyland N.K., Eggett D.L. (2013). Eating competence of college students in an introductory nutrition course. J. Nutr. Educ. Behav..

[9] Clifford D., Keeler L.A., Gray K., Steingrube A., Morris M.N. (2010). Weight Attitudes Predict Eating Competence among College Students. Fam. Consum. Sci. Res. J..

[10] Quick V., Byrd-Bredbenner C., Shoff S., White A.A., Lohse B., Horacek T., Colby S., Brown O., Kidd T., Greene G. (2016). Relationships of Sleep Duration With Weight-Related Behaviors of U.S. College Students. Behav. Sleep Med..

[11] Quick V., Shoff S., Lohse B., White A., Horacek T., Greene G. (2015). Relationships of eating competence, sleep behaviors and quality, and overweight status among college students. Eat. Behav..

[12] Quick V., Byrd-Bredbenner C., White A.A., Brown O., Colby S., Shoff S., Lohse B., Horacek T., Kidd T., Greene G. (2014). Eat, sleep, work, play: Associations of weight status and health- related behaviors among young adult college students. Am. J. Health Promot..

[13] Lohse B., Arnold K., Wamboldt P. (2013). Evaluation of About Being Active, an online lesson about physical activity shows that perception of being physically active is higher in eating competent low-income women. BMC Women Health.

[14] Psota T.L., Lohse B., West S.G. (2007). Associations between Eating Competence and Cardiovascular Disease Biomarkers. J. Nutr. Educ. Behav..

[15] Stotts Krall J., Lohse B. (2009). Interviews with Low-Income Pennsylvanians Verify a Need to Enhance Eating Competence. J. Am. Diet. Assoc..

[16] Satter E. (2007). Eating Competence: Nutrition Education with the Satter Eating Competence Model. J. Nutr. Educ. Behav..

[17] Stotts J.L., Lohse B. (2007). Reliability of the ecSatter Inventory as a Tool to Measure Eating Competence. J. Nutr. Educ. Behav..

[18] Lohse B. (2015). The Satter Eating Competence Inventory for Low-income persons is a valid measure of eating competence for persons of higher socioeconomic position. Appetite.

[19] Godleski S., Lohse B., Krall J.S. (2019). Satter Eating Competence Inventory Subscale Restructure After Confirmatory Factor Analysis. J. Nutr. Educ. Behav..

[20] NEEDs Center Protocol for the Use of the ecSatter Inventory 2.0. https://www.needscenter.org/wp-content/uploads/2019/09/ecSI-2.0-Usage-Protocol-2-1.pdf.

[21] De Queiroz F.L.N., Nakano E.Y., Ginani V.C., Botelho R.B.A., Araújo W.M.C., Zandonadi R.P. (2020). Eating competence among a select sample of Brazilian adults: Translation and reproducibility analyses of the satter eating competence inventory. Nutrients.

[22] BRASIL M., da S. Brasil M. (2020). VIGITEL 2019, Vigilância de Fatores de Risco e Proteção Para Doenças Crônicas por Inquerito Telefônico.

[23] IBGE IBGE—Instituto Brasileiro de Geografia: Pesquisa Nacional por Amostra de Domicílio Contínua (PNAD Contínua). http://www.ibge.gov.br/estatisticas-novoportal/sociais/educacao/1727-pnad-continua.html.

[24] World Health Organization WHO Mean Body Mass Index (BMI).

[25] Da Coqueiro R.S., Borges L.J., Araújo V.C., Pelegrini A., Barbosa A.R. (2009). Medidas auto-referidas são válidas para avaliação do estado nutricional na população brasileira?. Rev. Bras. Cineantropom. Desempenho Hum..

[26] Tilles-Tirkkonen T., Outi N., Sakari S., Jarmo L., Kaisa P., Leila K. (2015). Preliminary Finnish measures of Eating Competence suggest association with health-promoting eating patterns and related Psychobehavioral factors in 10–17 year old adolescents. Nutrients.

[27] Hargreaves S.M., Araújo W.M.C., Nakano E.Y., Zandonadi R.P. (2020). Brazilian vegetarians diet quality markers and comparison with the general population: A nationwide cross-sectional study. PLoS ONE.

[28] Okoli C., Pawlowski S.D. (2004). The Delphi method as a research tool: An example, design considerations and applications. Inf. Manag..

[29] Conti M.A., Scagliusi F., Queiroz G.K.O., Hearst N., Cordás T.A. (2010). Cross-cultural adaptation: Translation and Portuguese language content validation of the tripartite influence scale for body dissatisfaction|Adaptação transcultural: Tradução e validação de conteúdo para o idioma Português do modelo da Tripartite Influe. Cad. Saude Publica.

[30] Pratesi C.P., Häuser W., Uenishi R.H., Selleski N., Nakano E.Y., Gandolfi L., Pratesi R., Zandonadi R.P. (2018). Quality of life of celiac patients in Brazil: Questionnaire translation, cultural adaptation and validation. Nutrients.

[31] Farage P., Zandonadi R.P., Ginani V.C., Gandolfi L., Pratesi R., de Medeiros Nóbrega Y.K. (2017). Content validation and semantic evaluation of a check-list elaborated for the prevention of gluten cross-contamination in food services. Nutrients.

[32] Evans J.R., Mathur A. (2005). The value of online surveys. Internet Res..

[33] IBGE Instituto Brasileiro de Geografia e Estatistica PNAD Contínua TIC 2017: Internet Chega a três em Cada Quatro Domicílios do País. http://agenciadenoticias.ibge.gov.br/agencia-sala-de-imprensa/2013-agencia,-de-noticias/releases/23445-pnad-continua-tic-2017-internet-chega-a-tres-em-cada-quatro-domicilios-do-pais.

[34] Streiner D.L., Streiner D.L. (2003). Starting at the Beginning: An Introduction to Coefficient Alpha and Internal Consistency. J. Pers. Assess..

[35] Lohse B., Krall J.S., Psota T., Kris-Etherton P. (2018). Impact of a Weight Management Intervention on Eating Competence: Importance of Measurement Interval in Protocol Design. Am. J. Health Promot..

[36] Järvelä-Reijonen E., Karhunen L., Sairanen E., Rantala S., Laitinen J., Puttonen S., Peuhkuri K., Hallikainen M., Juvonen K., Myllymäki T. (2016). High perceived stress is associated with unfavorable eating behavior in overweight and obese Finns of working age. Appetite.

[37] Martins C.A., Machado P.P., da Costa M.L.L., Levy R.B., Monteiro C.A. (2019). Parents’ cooking skills confidence reduce children’s consumption of ultra-processed foods. Appetite.

[38] Hartmann C., Dohle S., Siegrist M. (2013). Importance of cooking skills for balanced food choices. Appetite.

[39] Reicks M., Trofholz A.C., Stang J.S., Laska M.N. (2014). Impact of Cooking and Home Food Preparation Interventions Among Adults: Outcomes and Implications forFuture Programs. J. Nutr. Educ. Behav..

[40] Jarpe-Ratner E., Folkens S., Sharma S., Daro D., Edens N.K. (2016). An Experiential Cooking and Nutrition Education Program Increases Cooking Self-Efficacy and Vegetable Consumption in Children in Grades 3–8. J. Nutr. Educ. Behav..

[41] McGowan L., Caraher M., Raats M., Lavelle F., Hollywood L., McDowell D., Spence M., McCloat A., Mooney E., Dean M. (2017). Domestic cooking and food skills: A review. Crit. Rev. Food Sci. Nutr..

[42] Burton E.T., Smith W.A. (2020). Mindful Eating and Active Living: Development and Implementation of a Multidisciplinary Pediatric Weight Management Intervention. Nutrients.

[43] Kuijer R.G., Boyce J.A. (2014). Chocolate cake. Guilt or celebration? Associations with healthy eating attitudes, perceived behavioural control, intentions and weight-loss. Appetite.

[44] Davidson D.J., Freudenburg W.R. (1996). Gender and environmental risk concerns: A review and analysis of available research. Environ. Behav..

[45] Ministério do Planejamento, Orçamento e Gestão Instituto Brasileiro de Geografia e Estatística—IBGE POF—Pesquisa de Orçamentos Familiares. https://biblioteca.ibge.gov.br/visualizacao/livros/liv50063.pdf.

[46] Krall J.S., Lohse B. (2010). Cognitive testing with female nutrition and education assistance program participants informs validity of the satter eating competence inventory. J. Nutr. Educ. Behav..

[47] Clifford D., Ozier A., Bundros J., Moore J., Kreiser A., Morris M.N. (2015). Impact of Non-Diet Approaches on Attitudes, Behaviors, and Health Outcomes: A Systematic Review. J. Nutr. Educ. Behav..

[48] Tilles-Tirkkonen T., Aittola K., Männikkö R., Absetz P., Kolehmainen M., Schwab U., Lindström J., Lakka T., Pihlajamäki J., Karhunen L. (2020). Eating competence is associated with lower prevalence of obesity and better insulin sensitivity in finnish adults with increased risk for type 2 diabetes: The stopdia study. Nutrients.

[49] World Health Organization (2003). Diet, nutrition and the prevention of chronic diseases. World Health Organ. Tech. Rep. Ser..

[50] Vartanian L.R., Schwartz M.B., Brownell K.D. (2007). Effects of Soft Drink Consumption on Nutrition and Health: A Systematic Review and Meta-Analysis. Am. J. Public Health.

[51] Ministério da Saúde (2019). Guia Alimentar Para a População Brasileira.

